# Smart Meter Data Collection Using Public Taxis

**DOI:** 10.3390/s18072304

**Published:** 2018-07-16

**Authors:** Kabeya Gilbert Ngandu, Khmaies Ouahada, Suvendi Rimer

**Affiliations:** Department of Electrical and Electronic Engineering Science, University of Johannesburg, Johannesburg 2195, South Africa; kouahada@uj.ac.za (K.O.); suvendir@nedbank.co.za (S.R.)

**Keywords:** data mule, smart meter, public taxis, wireless sensor network

## Abstract

The advent of wireless sensor networks (WSN) has opened up an array of applications. Due to the ad-hoc nature of WSN and the small size of wireless nodes, multiple system configurations are possible. In order to collect data from WSN, some systems utilize static nodes with a network setup that consists of multiple relays to facilitate the dissemination of data to a gateway. Other WSN architectures consist of a mixture of static and mobile nodes. Mobile nodes are able to collect data from the WSN when in close proximity to a static node. Such nodes are referred to as data mules. Data mules presents multiple advantages including the improvement of the network life as communication usually takes place via a single hop. In order to collect smart meter data, we propose the usage of mini-bus taxis carrying a data collector node as an alternative to traditional GSM models where data collected is directly uploaded from a data concentrator to a server. Using the vast network of mini-bus taxis in South Africa, data collection in areas lacking GSM network will be possible. This paper will attempt to present all the relevant parameters required for such data collection scheme to be successful.

## 1. Introduction

According to Statistics South Africa, 26% of municipal revenue in the last quarter of 2017 came from the sale of electricity [1]. This constitute the second largest source of revenue for municipalities. Given the importance of electricity sales revenue, great effort should be undertaken to correctly bill electricity consumers. Unfortunately, the current system is plagued with billing errors and a large portion of consumers are still billed base on electric consumption estimation rather than accurate meter readings. In order to accurately bill consumers, advance metering infrastructures (AMI) have been deployed in various areas. Smart meters used in AMI are able to periodically upload cumulative energy consumption and other data such as tamper alarms. The large amount of data collected can be analysed and used as an input to a larger system that is able to predict the consumption trend and energy demand. This system is called a smart grid. AMI is an extension of the early Automated Meter Reading (AMR) systems which were only able to communicate in a single direction. The typical components of an AMI are: the end user device; the communication modules and the meter data management system [2]. End user devices are generally classified as electricity meters, fluid meters (gas and water meter) and thermal meters [3].

**Figure 1:**
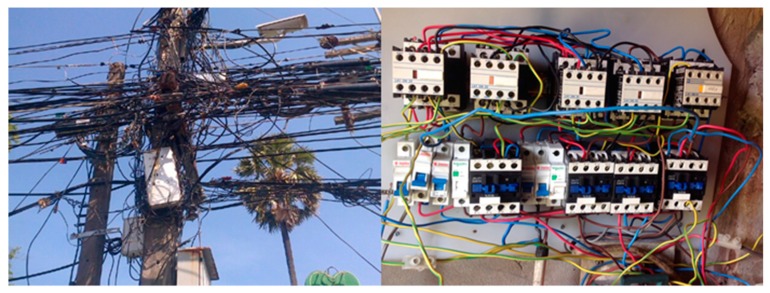
Illegal connections and Inadequate Setup.

Current smart metering techniques involves uploading data to a cloud server by using the GSM network. Data from multiple meters are transmitted to a data concentrator via multiple communication channels including power line communication (PLC), ZigBee, Ethernet, Wireless Meter Bus (WMBS) and more. Depending on the medium that needs to be measured, the best communication channel will be used. Fluid meters in the recent years are generally fitted with battery powered WBMS communication module that transmits meter readings wirelessly to a data concentrator. The batteries of these devices can last more than 10 years [4]. Amongst the multiple communication channels used in AMI for electricity, PLC has gained a lot of traction due to its simplicity and cost-effective deployment. Using PLC modems, data can be transmitted to a PLC concentrator through the existing power lines. The concentrator will then upload the data to a remote server through the GSM network. Three types of communication scheme exist in PLC namely: ultra-narrow band PLC (UNPLC), narrow band PLC (NPLC) and broadband over power lines (BPL) [5]. Ultra-narrow band operates between 125 Hz and 300 Hz. The bit rate at this frequency can reach up to 100 bps. Narrow band systems operate at frequencies between 3 kHz and 500 kHz. Ultra-narrow and narrow band PLC systems are able to communicate for several kilometres with narrow band systems able to transmit data at rates higher than 300 kpbs [6]. This makes NPLC suitable for several remote monitoring systems including smart metering. The BPL communication operating frequency is between 1.8 and 100 MHz. At this high frequency, a significantly shorter distance of less than hundreds of meters is achieved. The biggest advantage of this frequency range is the high data rate of more than 100 Mbps. Using BPL modems, network cables could easily be replaced by existing power lines to distribute internet in an office. Despite the advantages of PLC, it must be noted that a very high level of noise in the channel and significant signal attenuation affects the throughput of such communication infrastructure. One of the cause of these adverse effect, is the large number of end user electronic devices that use switch mode power supply. In some areas, the prevalence of illegal power line connections and substandard electric installations could also play a significant role in the reliability of power line communication. Figure 1 depicts such a situation.

**Figure 2:**
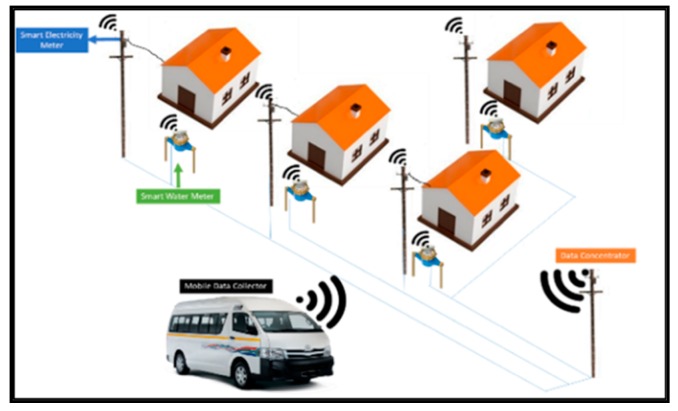
System Overview.

Our research objective is to design a system capable of collecting smart meter data using a mobile data collector fitted inside a moving mini-bus taxi. The mini-bus taxi (Kombi), shown in Figure 2, is a form of public transport where commuters are transported between taxi ranks via fixed routes. Taxi ranks are places where a group of taxis belonging to a certain taxi association regroup. From a taxi rank, mini-bus taxis’ routes are assigned to each driver. Mini-bus taxis in South Africa are currently the biggest form of public transportation in the country. Base on the national household survey of 2014, up to 68% of the population uses mini-bus [7]. On average, taxis operate from 5:00 a.m. to 7:00 p.m. At the taxi-rank, taxi line up and are dispatched as soon as they are full. According to Transactional Capital, a Johannesburg Stock Exchange listed financial service provider that have financed over 20,000 taxis, there are over 2600 taxi ranks. Each of their taxis cover on average 6500 km per month [8]. A maximum of 15 passengers can be seated in a Kombi. On the way to a taxi rank, commuters are allowed to exit the bus by signalling the driver. Drivers can also stop anywhere on the route to pick up passengers. As described in Figure 2, a taxi on his usual route could collect data from smart meter that are in communication range. Through various experiments, we will attempt to determine all the parameters required for such topology to yield a high packet transmission success rate. Such parameters could include the optimal speed for data collection, the technology that could be used, the communication range of the data collector under various environment and more. The solution that is described in this paper, focus on providing an alternative to the last communication leg between the data concentrator and a cloud billing server. The proposed solution will involve minimal alteration to existing AMI infrastructure. In some areas, GSM communication is very poor therefore a single hop communication between the passing taxi and the data concentrator could provide an additional method to transmit data to the relevant parties.

The paper is organized as follow: Section 2 contains a literature review covering the use of wireless sensor networks in advanced metering infrastructure. Section 3 is a description of the proposed system design, which is able to collect meter readings using public taxis. Section 4 describes the conducted experiments and presents the obtained results. Section 5 presents a discussion and analysis of the obtained results. Finally, Section 6 summarizes the conducted research and recommends suggestions for further extended work.

## 2. Related Work

In [9], the author compared PLC communication and WSN for automatic meter reading applications. The author pointed out the fact that both communication techniques are subject to high level of noise in the channels. The advantage of WSN over PLC is the fact that a reduction in the transmission range usually improves the communication while in PLC noise issues are more challenging to overcome. PLC system are not power constrained as compared to WSN infrastructures. In our case however, both the data mule and the data concentrator will be powered respectively by the vehicle’s battery and a power outlet. Furthermore, the communication technique used to upload smart meter data to the data concentrator is of no relevance as the main focus of the study is on the communication between the data concentrator and the data mule.

**Figure 3:**
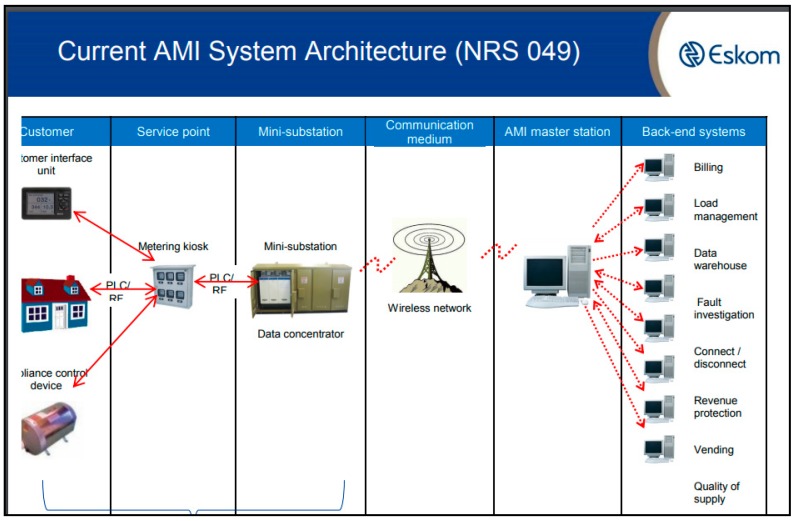
AMI Architecture.

ESKOM, the largest electricity provider in South Africa, published a series of specific requirements for smart electricity meters to ensure standardization of equipment across their network. The national standard NRS 049 depicted in Figure 3 [10], describes the current setup of most AMI systems in South Africa. The communication between the metering kiosk and the mini-substation is achieved through either PLC or wireless communication. With public transport passing daily in front of mini-substations, our system will collect meter readings directly from the metering kiosk or the mini-substation. This will ensure that some data is available during the billing cycle in case there is no connectivity between the data concentrator and the AMI master station.

The message transfer between a client system and a smart meter is achieved using the specification proposed by the standard transfer specification association (STS). This standard was initially developed by ESKOM to securely transport authorization message or token between point of sales and meters. It is currently used to securely communicate with a specific meter and also enables interoperability between multiple STS device from different manufacturer. STS token can be loaded on the meter via a keypad or a port. A token is not only an instruction to load credit but it can also be an instruction to view tamper status and more. This standard defines the application layer for smart meters. In the revised standard published by ESKOM, they proposed a communication interface called virtual token carrier (VTC) that enable secure serial communication with smart meter in order to load tokens, read cumulative energy consumed, view tamper status and more. The transmission speed of the VTC must take place at 2400 bits per seconds. To star transmission with the smart meter, a 5 bytes identification message must be sent by the client system. Once the communication is established, a specific command to read/write from/to a meter is sent and an acknowledgement or a data message is expected from the client. This process is described in detail in [11].

Abrar et al. [12] presented a simulation of a ZigBee based AMI network able to collect real time load data, theft detection and line loss. The proposed architecture involved having a local control centre (LCC) placed at transformer and smart meter fitted with ZigBee modules. The LCC was able to upload the local meter readings to a master data management centre via GSM. It was shown that reliable communication was possible but the author stressed the need for robust security to be implemented.

In [13], Karyemsetty et al. experimented with the possibility of transmitting GPS location and speed of moving vehicles to a static road side unit (RSU). The communication was effected using ZigBee enabled devices. The road side unit was setup as a coordinator in broadcast mode with association bit active. The road side unit broadcasted a message to all the vehicle in order to receive their data. The author noted a deterioration of communication between the vehicle and the RSU as the vehicle speed started increasing.

The concept of using mobile nodes to improve communication in areas with communication holes has been extensively discussed before. In [14], the author proposes a routing technique called Data Pre-forwarding (DPF). This scheme consists of forwarding data to nodes with high probability of contact with the mobile sink. The transmission of data to a data concentrator with a better chance of contact with the passing mobile sink is effected whether the mobile sink is in vicinity or not. When a data mule navigates through a wireless sensor network, the contact time with the source node can be as short as a few seconds depending on the speed of the mobile node. Therefore, the author was able to prove that sending data to a high resource data concentrator able to be in contact with a data mule can improve the data collection success.

An opportunistic routing scheme that can give us insight into our research topic is the concept of routing messages in a WSN base on the location of the mobile node but more importantly based on the social behaviour of the node carrier. Enes et al. [15] used the frequent visit of specific nodes to identify the best carrier nodes or data mule and set frequently visited locations as hubs. After determining the best carrier, the routing table is updated to optimise message dissemination in the network. A hub can be formed by a group of nodes that often visit a specific location. An example of social hub can be a group of mobile phones that are often in a specific area and another group of mobile phones that are often at a taxi rank. If a message needs to be carried to that area, it will be forwarded to a node that is known to be travelling from a taxi rank to the target area at specific time intervals. In this approach node context are formed using the geographical position of the node, the travel history and the historic of the node encountered.

Mini-bus taxis in South Africa have specific routes they travel throughout the day. The trajectory based statistical forwarding (TSF) could be an ideal infrastructure to vehicle (I2V) routing algorithm that could yield high packet success rate. TSF forwards packets through multiple-hops to a rendezvous point where the vehicle is expected to pass. In TSF, the vehicle trajectory is known by the stationary data concentrator. The data concentrators are placed at position where the vehicle is expected to slow down such as an intersection or a tollgate. Jeong et al. [16] focused on improving the delivery delay to the vehicle. They concluded that, higher vehicle speed yields a smaller delivery delay. Based on the success of TSF, an extension of the algorithm focusing on message delivery to a group of vehicles rather than a single vehicle was developed in [17]. Trajectory-based I2V group message delivery protocol (eTGMD) correctly states that due to high density of vehicles in urban areas, there is a probability of multiple vehicles being at the same intersection. eTGMD is able to achieve high delivery ratio even in high density area.

Mulla et al. [18] looked at the similarities between WSN and AMI. As described in their paper, a meter is simply a node that serves as an energy interface for building area networks and similar networks. They also analysed the specific challenges of using ZigBee as part of an AMI, where standard AMI can collect large amount of data from each meter. Their paper shows that due to the low data rate of ZigBee, IPV6 based WSN might be better suited to handle larger packets. Another issue encountered with WSN is the fact that wireless node identification can be random hence communication with a specific node might be challenging. Meters should be tamper resistant therefore wireless sensor nodes should be able to resist attacks that could affect the operations of the system. Mulla et al. [18] also pointed out the need to secure smart meter encryption keys in the wireless communication. These challenges can be resolved by introducing a robust application layer algorithms.

Kumaar et al. [19] discussed the issue of the speed of data collection from a mobile sink where the path and the collection time are limited. They referred to the problem as maximum data gathering motion planning of a mobile sink within a fixed time deadline or (MDMPMS). In order to reduce the data collection delay, Kumaar et al. have developed a route planning and optimization algorithm for the mobile sink. This algorithm simply specifies the speed of the mobile sink at a specific time. Kumaar et al. found that the data collection success was proportional to the number of node in the network.

## 3. Proposed Solution

As described in the introduction, the aim of our research is to determine all the parameters required for a data mule fitted on a mini-bus taxi to be able to collect meter readings. After conducting an extensive literature review of relevant documents, this section will describe a system able to achieve our objective. 

### 3.1. System Architecture Description

In order to determine the consumption of a meter for a specific month, at least two readings are required. One at the beginning of the month and the second at the end of the month. The system we are proposing will collect the following information from each meter:

- Meter Serial Number (7 bytes)
- Total Cumulative Unit Consumed (4 bytes)
- Tamper Status (2 bytes)
- Available Credit (4 bytes)
- Date Stored (4 bytes)

This information will be recorded in the data concentrator and will be transmitted to the passing data mule whenever possible. As described in the literature review, communication with the smart electricity meter can be performed through the VTC port.

**Figure 4:**
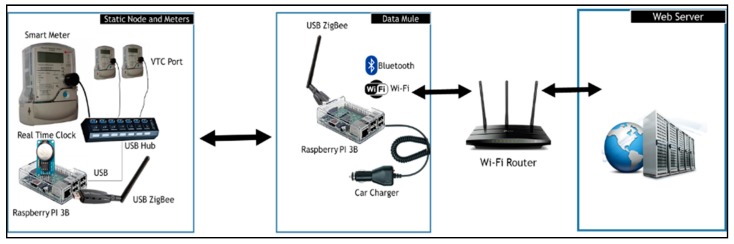
Proposed Architecture for Low Number of meters.

In order to collect relevant data from smart electricity meters, we propose the architecture in Figure 4. The static node will be a Raspberry PI 3B connected to a ZigBee antenna. The choice of a Raspberry Pi 3B is due to the fact that the device incorporates all that we envisioned a data mule should have. It has a built-in Wi-Fi and Bluetooth module and include multiple USB ports that will be used to connect a ZigBee module and optical data readers. Once the data mule is in range, it will connect to the wireless sensor network, collect the meter data and store it. The data mule will then connect to a free Wi-Fi network such as the TshWi-Fi (Tshwane Free Wi-Fi) or a Wi-Fi installed at the taxi rank. Once the data mule is connected, it will upload the data to a webserver where the data will be made available to the metering department or company.

**Figure 5:**
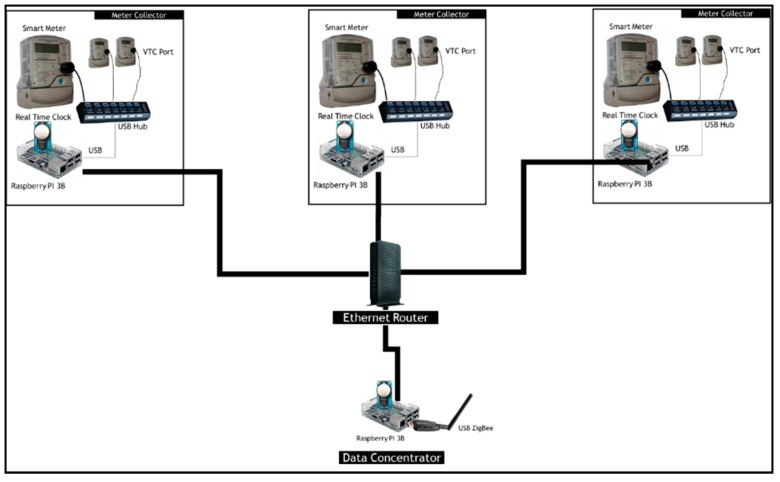
Proposed Architecture for large Scale Roll Out.

The architecture proposed in Figure 4, will be limited to the number of USB optical port that can be supported by a host system. The maximum number of USB device that can be supported by a single host system is 127 [20]. Given, that our host system is a resource limited Raspberry PI, the proposed architecture will struggle to operate reliably with a high number of meters connected to the same Raspberry PI. To solve this issue, multiple replica of this architecture could be setup with different PAN IDs. One of the issues that could emanate from such a setup is the inability of the data mule to connect to the different PANs since the ZigBee antennas might be next to each other. This issue is commonly referred to as a hidden terminal problem. In the light of issues described earlier, we propose a scalable architecture of the data concentrator as depicted in Figure 5.

The system proposed in Figure 5, includes meter collectors that will store meters data and upload it to the data concentrator through a wired Ethernet router. The choice of using wires between the meter collector and the data concentrator is to reduce interferences that Wi-Fi communication could induce in the ZigBee channels. A meter concentrator will only be directly connected via USB to a small number of smart electricity meter that the Raspberry PI can reliably communicate with.

Although the proposed solution makes use of optical ports and Ethernet to gather data and store it in the Raspberry PI concentrator, any other communication methods including PLC could be used to transfer individual smart meter data to the data concentrator.

### 3.2. Data Concentrator

**Figure 6:**
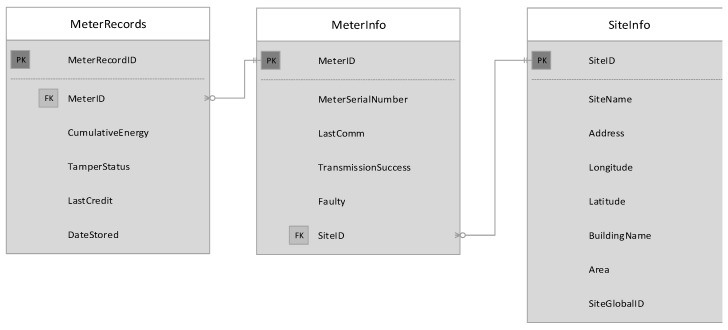
Data Collector Database Design.

As described earlier, two system architectures are proposed where the difference appears in the design of the data concentrator. In the first architecture, the data concentrator collects meter readings and directly communicates with the data mule. In the second architecture, the data concentrator includes a sub-concentrator that stores meter readings and sends the collected data to a main concentrator via Ethernet. The main concentrator will in turn forward the packets to the data mule. In both cases the data will be stored in a local MySQL database. The database will be structured as described in Figure 6. The database will have 3 tables described as follow:

- **SiteInfo**: This table will be used to store information regarding the location of the data concentrator. It will have a unique global site identification loaded during commissioning.
- **MeterInfo**: Once the meter serial number is retrieved from a specific meter it will be inserted to this table. If the meter number already exists, the existing data will be updated. As soon as serial communication is successful with a meter, the last communication date and time will be updated. The transmission success field is an integer that will be incremented every time a meter record is successfully transmitted to the data mule. Records to be send to the data mule will be sent sequentially therefore the transmission success field will help us determine which meters have the lowest transmission success rate and put those meters in first position when data need to be send to the data mule. The sorting process will occur every time a data collection request is received. If the system fails to communicate with the meter for more than a day the faulty field will be set to true.
- **MeterRecords**: this table will store the cumulative energy consumed, the tamper status, the last credit value and the date it was stored.

The packet represents the data structure required to build a basic billing system. Using two records, the billing system will be able to compute the energy consumption for the period between the two records. In this packet, the SiteID is not required as this value can be obtained from the billing server using the meter number. The MessageID is also optional and only included for future improvement of the routing process.

### 3.3. Data Concentrator Design for Low Number of Meters

The data concentrator for residential complex with a low number of meters will be composed of the following components:

- Raspberry Pi 3B: This module will be the central processing that will store and issue commands to the meters on a regular basis and also upload the data stored to a webserver when it is required.
- USB ZigBee Module: This module will be the ZigBee network coordinator for a specific building or residential complex. It will create a PAN that the data mule will connect to and collect the meter readings.
- USB optical cable: Electrical smart meters offer a VTC port which is an optical port that provide serial communication with the meter. A USB optical cable will be used to communicate with the meter.
- Real Time Clock Module (RTC): the RTC is a battery powered module that can provide accurate time to device connected to it. Accurate time is crucial to our system. Data collected from the meters will be stored with a date and time.
- USB Hub: to help connect to the multiple smart meters.

**Figure 7:**
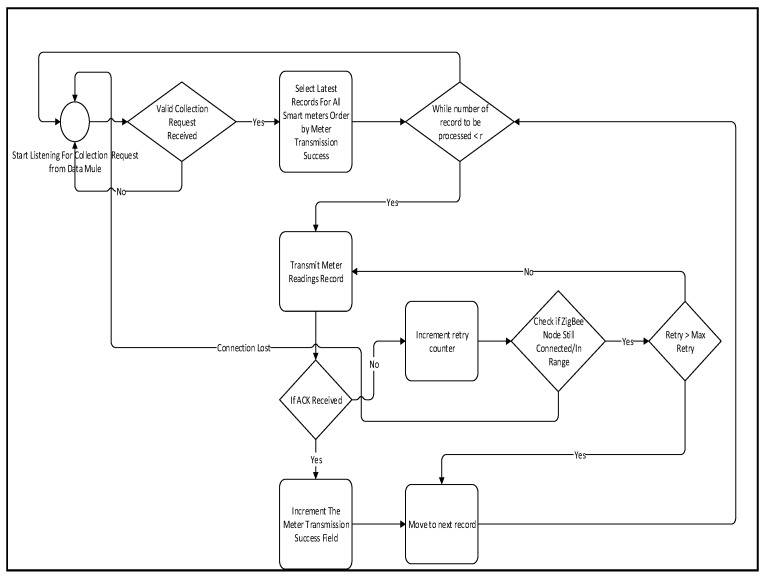
Data Collector Transmission Flow Chart (Low number of meters).

**Figure 8:**
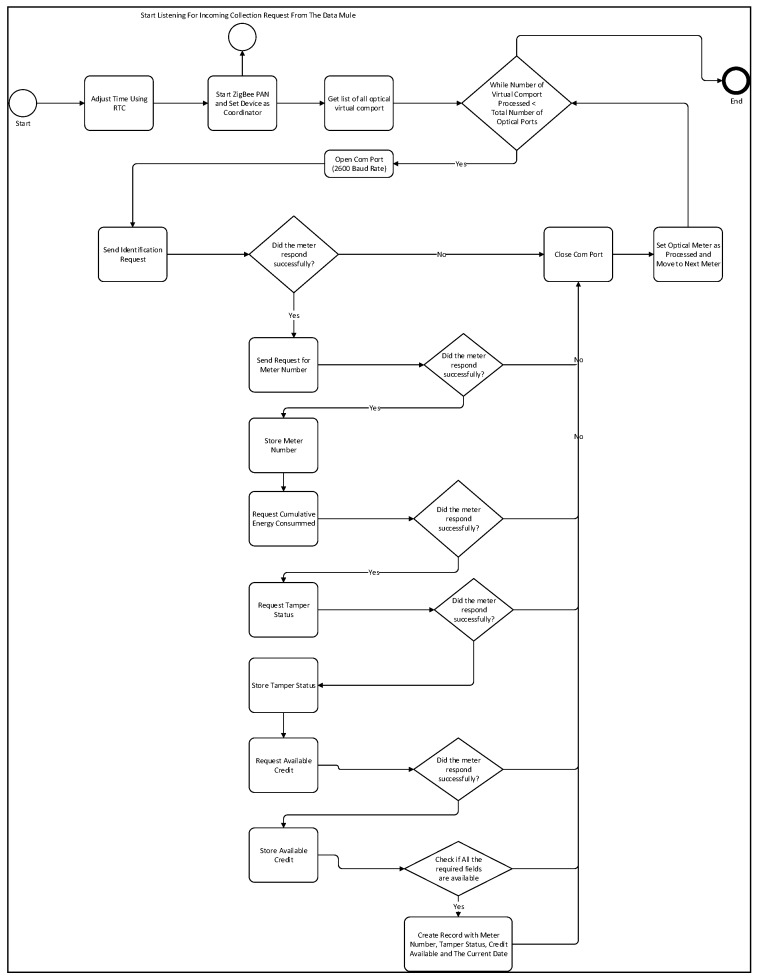
Data Concentrator Meter Reading Flow Diagram (Low number smart meters).

The system will start by adjusting the device current date and time by communicating with the real-time clock module. Once the time is adjusted, the data concentrator will create a ZigBee PAN and setup the node to be a coordinator. The data concentrator transmission process described in Figure 7, will start as soon as the ZigBee PAN has been established. Once a USB optical port is connected to the Raspberry PI, the device creates a virtual serial communication port with a port name. As described earlier, in order to communicate with the meter through the VTC port, a communication baud rate of 2600 must be used. The next step will consist of listing all the optical port’s virtual comport address. The system will then proceed by opening each serial port one at the time. For each optical port, the system will first send an identification message and wait for a response. If the identification response is successful, the system will proceed by sending a request for the meter to provide its serial number. The cumulative energy consumed, the tamper status and the available credit will then be collected. Once all the required data for a single meter has been collected, we will store that information in a local MySQL database. The data concentrator flow diagram is described in Figure 8.

The concentrator will start by opening the serial port associated with the USB ZigBee module. Once the port is open, it will start listening for incoming packets. If a valid data collection request is received, the system will retrieve a list of meter reading records ordered by the meter transmission success. The meter transmission success is an integer that gets incremented every time a successful transmission takes place. Ordering records by meter transmission success will give a fair chance to all the meters that have not transmitted enough records to always be on top of the transmission queue. An additional process to check if the meters with low transmission success are faulty will take place in order to prevent unnecessarily prioritizing a faulty meter. This process will simply check when last the meter readings were successfully collected. If the last data collection from the meter took place more than a day ago, a faulty flag will be raised and stored in the MeterInfo table. In case there is no transmission acknowledgement, the system will first check if the node is still connected to the network. If it is not connected, the process will go back to the beginning a wait for a collection request.

### 3.4. Data Concentrator for Large Meter Deployment

For buildings or residential complex with more than 100 m, the system architecture in. will be more suitable. This setup will include a main concentrator that will collect data from sub-concentrators. The main concentrator will run an instance of Apache server that will have a PHP API able to receive HTTP packets and store the relevant information in a MySQL database with the same structure described in Figure 6.

**Figure 9:**
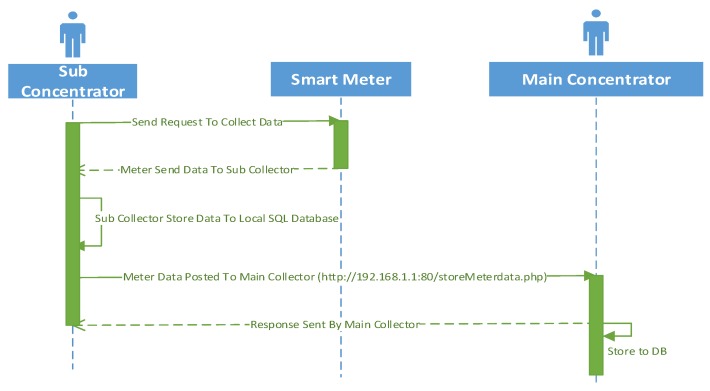
Sequence Diagram for Large Scale Data Concentrator.

The sequence diagram for large scale setup is depicted in Figure 9. The data concentrator transmission process will be exactly the same as the low meter setup. The particularity of this architecture is only the fact that the data collection from the smart meter has been relegated to sub-concentrators and the transmission to the data mule only occurs at the main concentrator.

### 3.5. Data Mule

The data mule will be setup as a ZigBee router able to join any PAN in its transmission range. Once the data mule is connected, it will send a request to collect data. Once the request is received by a concentrator, it will start broadcasting meter readings to all authorized data mules. The collector will keep sending data to the data mule until it stops accepting new messages by sending a NACK message or leaving the PAN.

**Figure 10:**
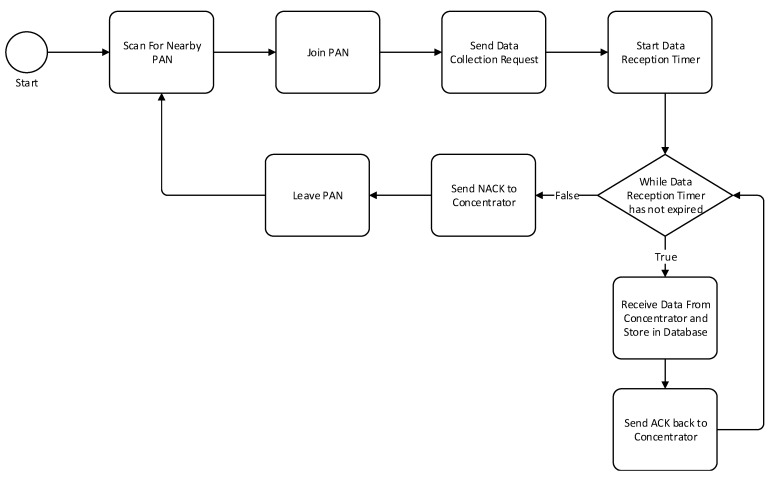
Data mule flow diagram.

**Figure 11:**
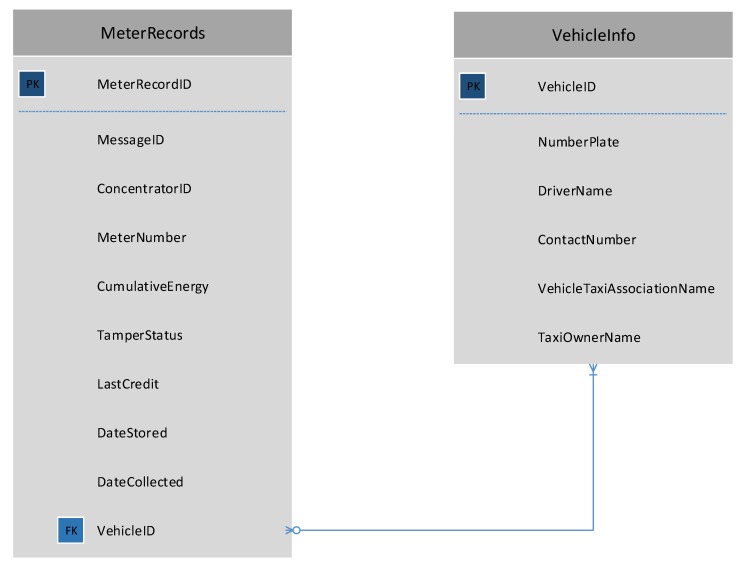
Data Mule Database.

**Figure 12:**
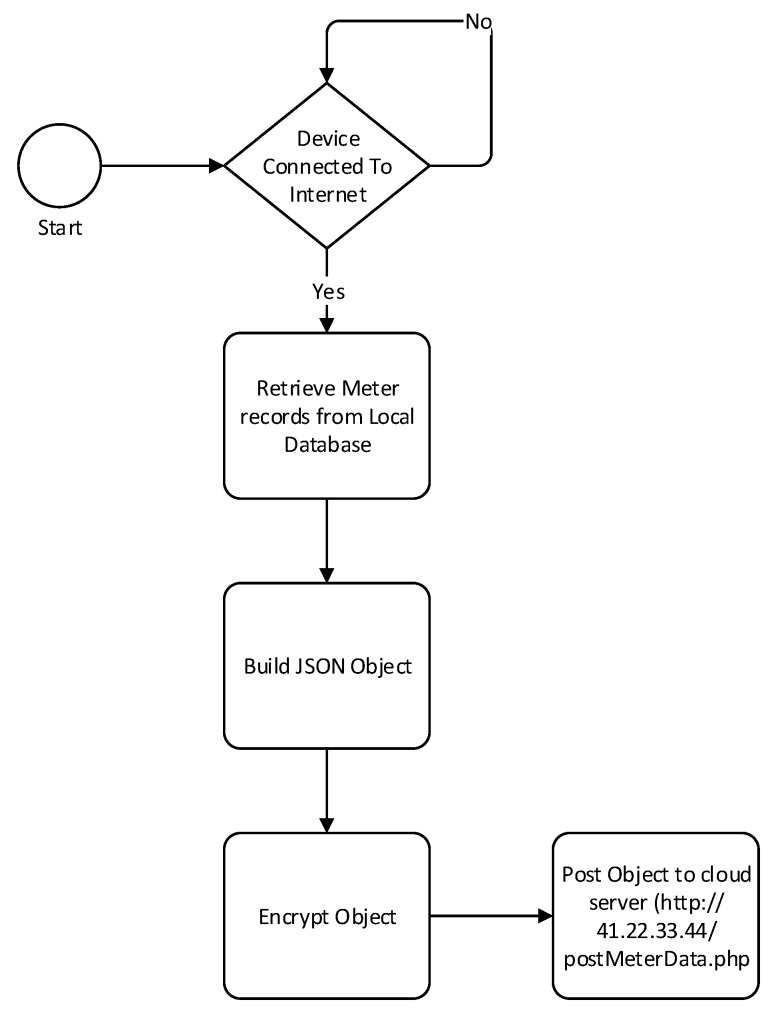
Data mule to Billing Server Upload.

The first step for the data mule collection process is to scan for nearby ZigBee PAN, once a PAN is in range, the data mule will join the network. Once in the network the device will send a data collection request to the data concentrator. As soon as the message is received by the coordinator, a data reception timer will start. While the data reception timer has not expired, the data mule will keep sending meter records and it will be stored in a MySQL database. This process is described in Figure 10. The database includes two tables: MeterRecords and VehicleInfo as described in Figure 11. The first table will be used to store all the meter records collected from all the data concentrator that the data mule managed to interact with. The vehicle information table is used to keep a record of who owns the vehicle, the driver name and other details. Data collected from the data mule can be uploaded to the billing server either through Wi-Fi or Bluetooth. The raspberry PI will be configured with a list of Wi-Fi access points that it can connect to. Once a Wi-Fi connection is established, a JSON object containing all the device records will be posted to the cloud server. This is depicted in Figure 12.

**Figure 13:**
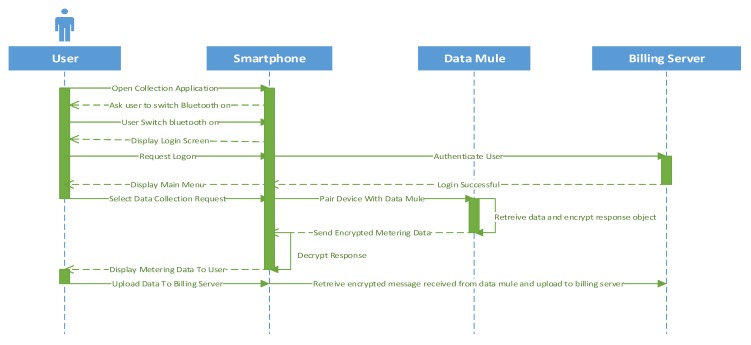
Sequence diagram for retrieving metering data via Bluetooth.

**Figure 14:**
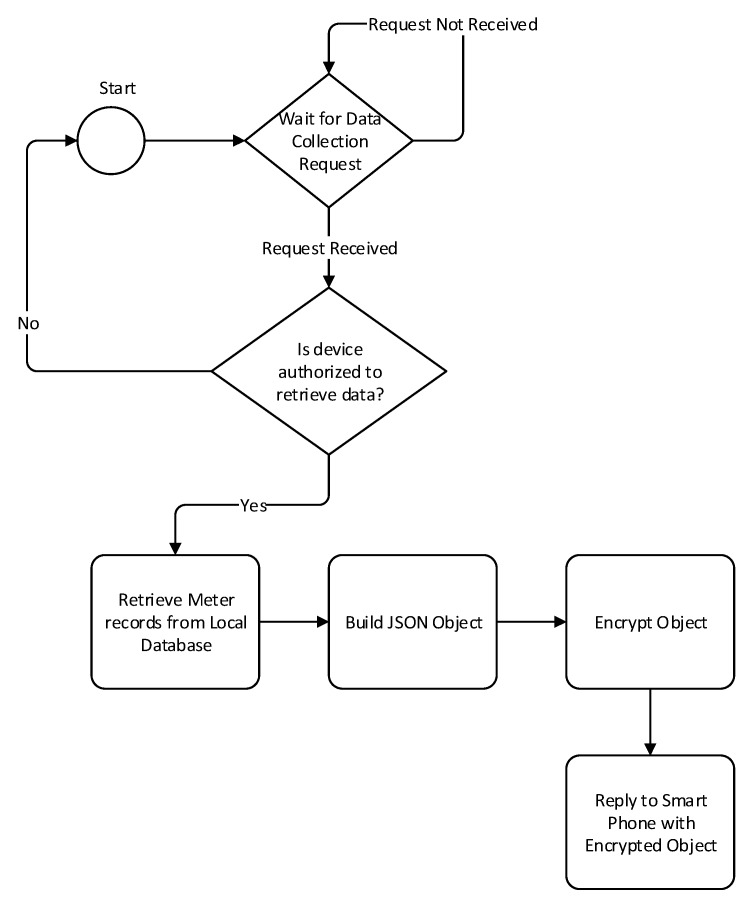
Data transmission from data mule via Bluetooth.

To collect metering data through Bluetooth, a mobile application will request to be paired with the data mule. Once the device is paired, it will send a data collection request in order to receive the metering data. The packet received could then be uploaded to the billing server. The sequence diagram depicting this process is illustrated in Figure 13. The steps taken by the data mule to send the data back to the smart phone are depicted in Figure 14.

## 4. Results

### 4.1. Wireless Sensor Node Mobility and Its Effect on Transmission Reliability

**Figure 15:**
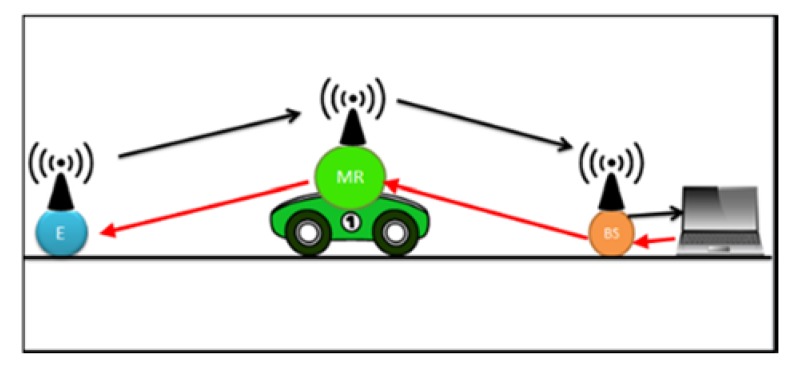
Mobile Router Experiment.

We begin our series of experiments by an experiment aimed at determining the effect of speed on the reliability of communication between wireless nodes in a real environment. In order to achieve our experiment objective, we placed three nodes as illustrated in Figure 15.

**Figure 16:**
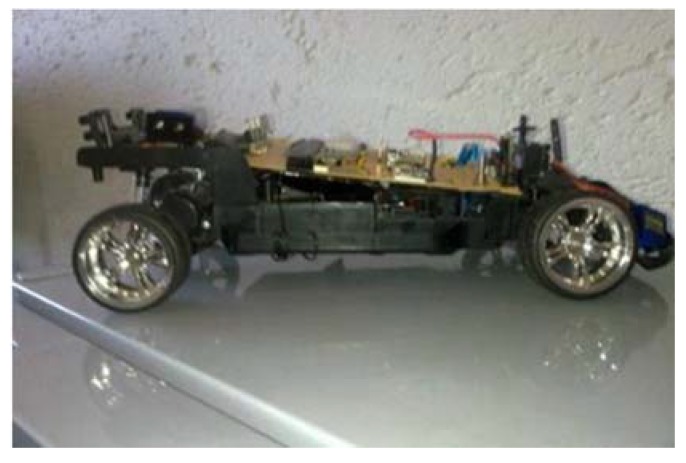
Mobile Router.

The endpoint and the base station were placed within line of sight. Since our aim was to determine the effect of the mobile relay motion speed on the communication link, the mobile relay was moved from the endpoint to the base station at various speeds while the endpoint was transmitting data. The following apparatus was used:

- Computer with XCTU software installed and a USB XBee S1 module containing an API coordinator firmware.
- One end point node composed of a Microchip 16F917 and an XBee S1 XB24-AWB-001 RF module with an End Point API firmware loaded.
- A toy car fitted with an XBee S1 module acting as a router node as illustrated in Figure 16. On this module, the receive PIN RX and the transmit PIN TX were shorted in order to immediately transmit the received message to the base station.

**Figure 17:**
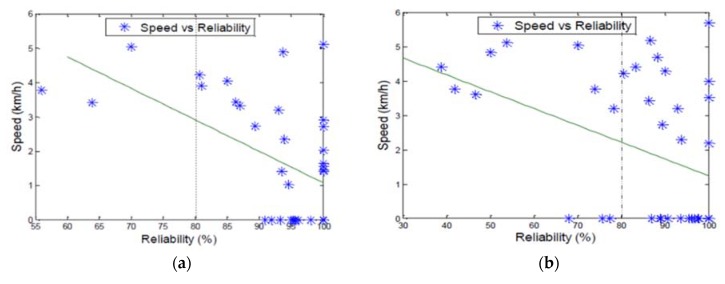
These figures depict the relationship between the speed and the reliability. (**a**) Speed vs. reliability 8 bytes. (**b**) Speed vs. reliability 16 bytes.

This experiment was conducted with a packet size of 8 bytes and a speed between 0 and a maximum of 6 km/h. The results of are depicted in Figure 17a.

These results show that the reliability of communication is higher at lower speed. As the node moves, higher multipath fading is induced in the wireless communication. It can cause the communication channel to frequently change. This instability is shown to have a detrimental effect in the communication.

The previous experiment was repeated with a lager message and the results are depicted in Figure 17b. By comparing the packet success rate below the 80% reliability threshold in Figure 17a,b, it is clear that Figure 17b has a higher number of unreliable packets. This is caused by the increased time required to clear the transmission buffer of the ZigBee device.

As the speed increases, there is a trend for the reliability to start deteriorating. We can see that the speed had greater effects on the reliability when the message size was increased. Since the message is larger, the packetization time has increased. Using the same data rate as the 8 bytes experiment will generate more errors. Decreasing the transmission rate while the mobile relay is moving could improve the reliability. In the context of our research, mini-bus taxis are prone to make multiple stops to either drop or pick-up passengers. At those points, the highest packet success rate will be achieved if the data concentrator is in range.

### 4.2. Determination of the Transmission Range and the Maximum Packet Size

The objective of this experiment is to determine the maximum transmission range of the ZigBee devices modelled in NS3. This experiment will also determine the maximum reliable packet size. For this experiment, we configured two ZigBee devices to communicate with each other and we gradually increased the distance between them until no packets were received. We also increased the size of the packet gradually to determine the packet size threshold.

**Figure 18:**
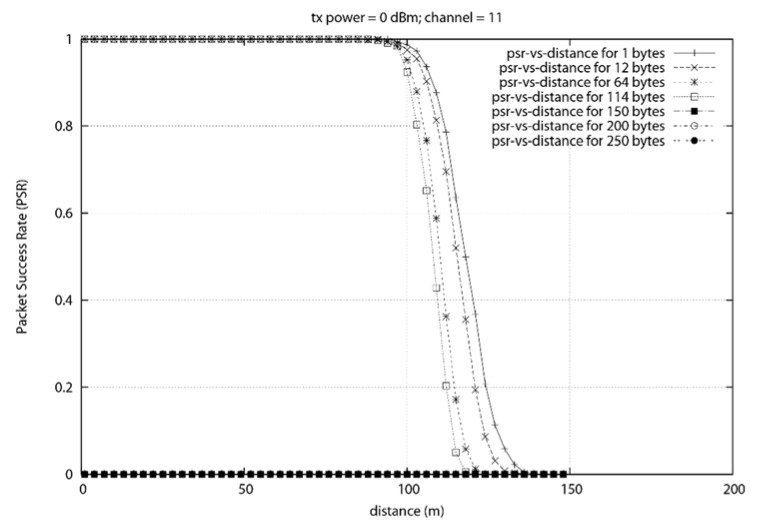
PSR vs. Distance with different packet size.

Using NS3, two ZigBee nodes were created. A thousand packets per seconds was transmitted at each position and the packet success rate was computed. It can be seen From Figure 18, that the smallest packet is able to be transmitted at the furthermost distance. We can clearly see that the transmission range is affected by the size of the packet. This corroborate with the result obtained in Section 4.1 where the PSR decreased when the packet size was increased.

This experiment shows that packets can be transmitted at a distance of up to 112 m with a success rate of more than 80%. It was also found that an increase in packet size slightly reduce the reliable transmission range. Since the data rate is constant a bigger packet will require more time to be transmitted. 

The reduction in the transmission range is mostly due to the buffer size of the nodes. Once the buffer is full, the device will start losing new incoming packets. The maximum packet size that yielded an 80% transmission success was found to be 114 bytes. The difference in transmission range between the 1 byte and the 114 bytes packet at the 80% PSR was found to be 9 m. This represents an 8% difference in transmission range. We can conclude that the packet size to be transmitted should be less than 114 bytes and the distance between the transmitter and receiver should be less than 112 m in order to ensure reliable transmission.

### 4.3. Analysis of Data Mule PSR Using Typical Taxi Speed

From Section 4.2, we were able to determine that the speed does indeed impact the reliability of the signal but due to physical constraint of the real environment we were unable to run test with a larger dataset (between 5 km/h and 120 km/h). This experiment through simulation will reveal the impact of speed on the communication success rate. We will also analyse how this data collection scheme will perform with different distances from the data concentrator to the road used by the minibus taxi. Based on our results we will attempt to derive a formula that would help determine the maximum distance between the data mule and the data concentrator. We will also determine under which condition the formula obtained will best work.

NS3, we have setup an environment with two nodes. We attempted to model an XBee-PRO with transmit power of 63 mW or 18 dBm. In order to simulate an urban environment, we used a Log-distance path loss model with loss exponent of 3.5. A loss exponent of 4 and above will be used for busy environment with buildings as indicated in [21].

**Figure 19:**
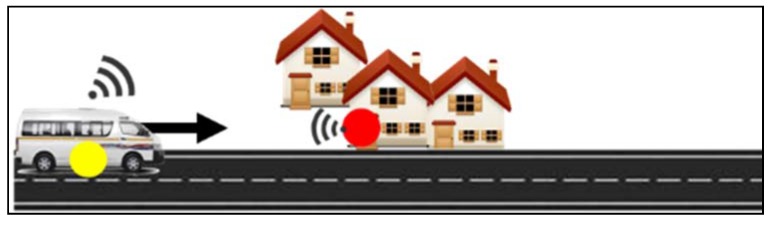
Experimental Setup.

Figure 19 illustrates a taxi moving toward a smart meter data concentrator. The taxi starts at position x = 0 m, y = 5 m (y is the distance from the meter concentrator to the road) and an elevation of z = 2 m (2 m being the height of the taxi if we assume the data mule antenna is on the vehicle’s roof). The end position is at position x = 1000 m, y = 5 m and z = 2 m. The meter concentrator is placed at position x = 250 m, y = 0 m and z = 2.4 m (The 2.4 m elevation represents an antenna placed on a wall).

In this series of experiments, the taxi node moves toward the meter data concentrator with speeds of 5 km/h, 60 km/h and 120 km/h. At each speed and position, a 64 bytes packet is transmitted 1000 times from the meter concentrator to the mobile collector. The success rate is then computed based on the number of packet received. This experiment was extended to multiple other speed in order to establish a meaningful relationship between the speed and the reliability of the packet success rate.

We also investigate the possibility of collecting data from an area with a high number of building such as Johannesburg central (Johannesburg is the business hub of South Africa). From a survey of building height using Google Earth Pro, we noticed that most buildings have a height below 150 m. Assuming the data concentrators are located on top of the buildings, we will analyse the effectiveness of our proposed data collection scheme.

Johannesburg central is known to have a large number of traffic lights and a high traffic throughout the day. In such area, we will use a maximum speed of 60 km/h and also determine the average speed during peak times and when there is no traffic. The path loss exponent used will be four. The average speed calculation will be affected by the high number of traffic lights in the area and the fact that mini-bus taxis have to stop to drop passengers.

**Figure 20:**
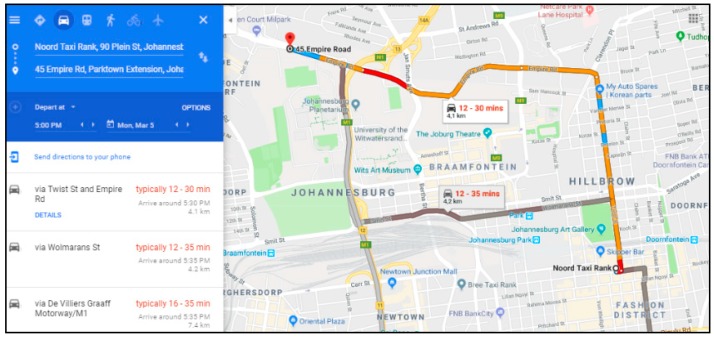
Traffic Data from Noord (suburb 1) Taxi Rank to Milpark (suburb 2) at 5:00 pm.

**Figure 21:**
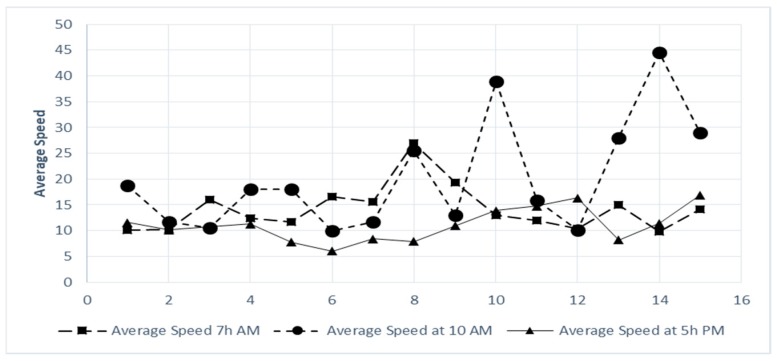
Comparison of Average Speed in Town.

We will compute this average speed by collecting the average time to travel a specific distance in and around Johannesburg town. The data was obtained by using Google Maps traffic data as described in Figure 20. This web application enables us to view traffic information from previous dates as well as specifying the time. Figure 21 depicts the average speed at 7:00 a.m., 10:00 a.m. and 5:00 p.m. It can be seen that the maximum average speed is 45 km/h. This slow speed is caused by the frequent stops the bus has to make.

**Figure 22:**
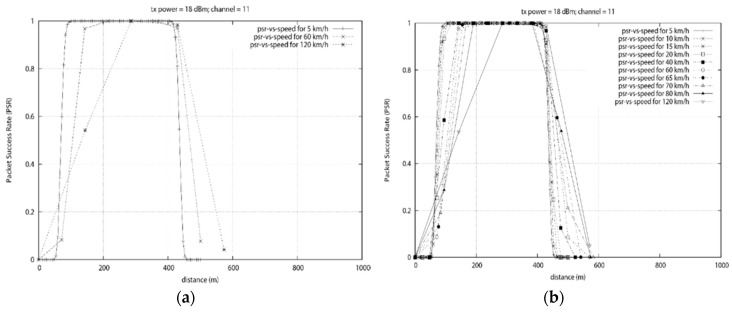
This figure depicts the Speed vs. Reliability in Residential Road. (**a**) Speed vs. reliability with concentrator at x = 250 m (**b**) Speed vs. reliability with multiple speeds.

In Figure 22a, the static data concentrator is placed at position x = 250 m. The mobile node travel toward it with different speeds while transmitting data. From Figure 20, the slowest speed provides the largest communication range above 80% reliability. Accurate communication is possible for all the speeds tested but the high PSR communication range decreases as the speed increases. As expected, the positions with the best PSR are around 250 m.

Figure 22b is an extension of results obtained in Figure 22a that will be used to derive a pattern or formula to represent the relationship between the speed and the communication range of the mobile node. As the speed increase, fewer data is collected by the data mule as it spends less time travelling in the communication range. One noticeable result is the fact that at a speed of 120 km/h, the first set of results with a PSR above 80% was obtained at position x = 285.6 m. This is 35.6 m after passing the data concentrator placed at x = 250 m. This is due to the slow data rate of the ZigBee device.

**Figure 23:**
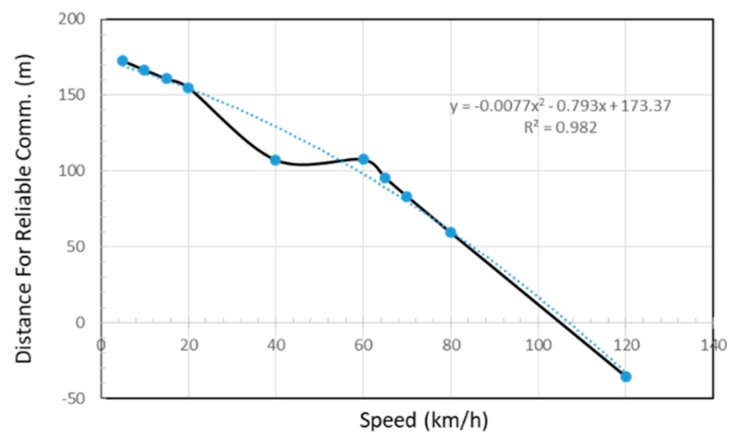
Speed vs. Distance for Reliable Communication (80% PSR).

Based on the various results obtained a relationship between the speed and the distance required for reliable communication is derived in Figure 23. We mapped the speed of the data mule and the distance x where 80% PSR is first achieved. From the Figure 23, it can be seen that if a data mule travels at a speed of 20 km/h, reliable communication will start when the vehicle is around 150 m from the data concentrator. When the data mule travels at 120 km/h, the vehicle will first pass the data concentrator positioned at 250 m and communicate reliability for a short period at position 285.6 m which is 35.6 m after the concentrator.

**Figure 24:**
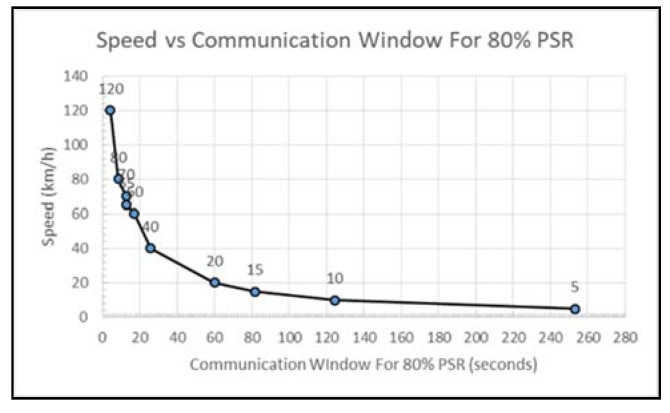
Speed vs. Communication Window for 80% PSR.

The relationship between the speed and the distance for reliable communication will help determine the optimum position a data concentrator should be placed based on the speed limit of the nearby road where the data mule is expected to travel. Once the data concentrator has been optimally placed, we need to determine how much time the data mule will have to communication with the concentrator based on the speed of the data mule. Figure 24, depicts the relationship between the speed and the communication window. At 20 km/h, the data mule will have 60 s before it leaves the reliable communication range. At speed of 60 km/h communication must take place under 20 s. On highways, where the speed limit is 120 km/h, less than 10 s will be available.

**Figure 25:**
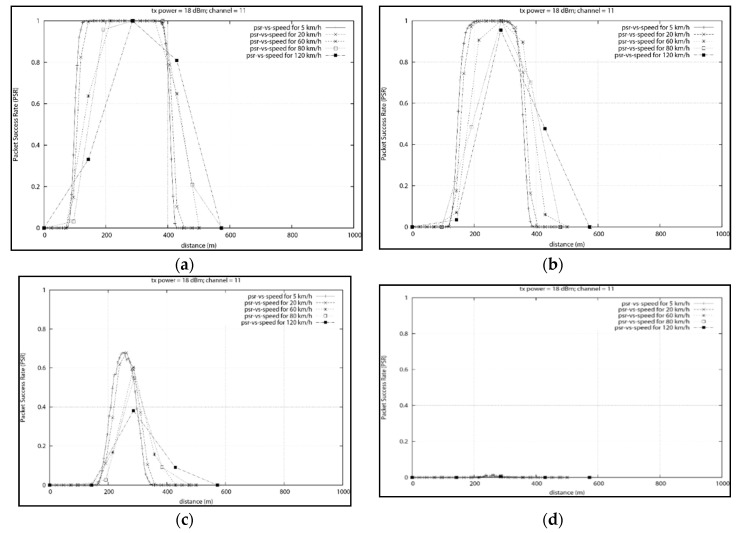
PSR vs. Speed with concentrator at position x = 250 m and various y positions (**a**) y = 100 m (**b**) y = 150 m (**c**) y = 180 m (**d**) y= 200 m.

In Figure 25a, the data concentrator is still placed at position x = 250 m but the vehicle passes the concentrator at y = 100 m. The y position is increased from 100 m to 200 m. As illustrated in Figure 25, the 80% PSR communication is possible for up to 150 m. As depicted in Figure 25c, at position y = 180 m from the mobile collector, only a maximum of 65% PSR is possible. Any position higher than 180 m will yield poor results as seen in Figure 25d.

**Figure 26:**
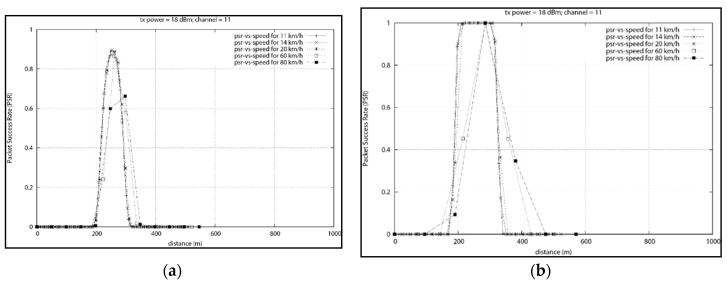
Speed vs. reliability for concentrator on buildings in busy area (4.0 path loss exponent) (**a**) 60 m high, (**b**) 90 m high.

In Figure 26a,b, the data concentrator is placed on top of buildings with different heights (60 m and 90 m). In order to simulate an environment with a large number of building and obstacles, a path loss exponent of 4 was used. The distance between the building and the passing vehicle was 30 m. The 80% PSR communication range has become very narrow compared to a setup were both the concentrator and data mule are at the same height.

When the building height is increased to 90 m, Figure 26b shows that the 80% PSR communication is possible for a shorter distance compared to the 60 m high building in Figure 26a.

**Figure 27:**
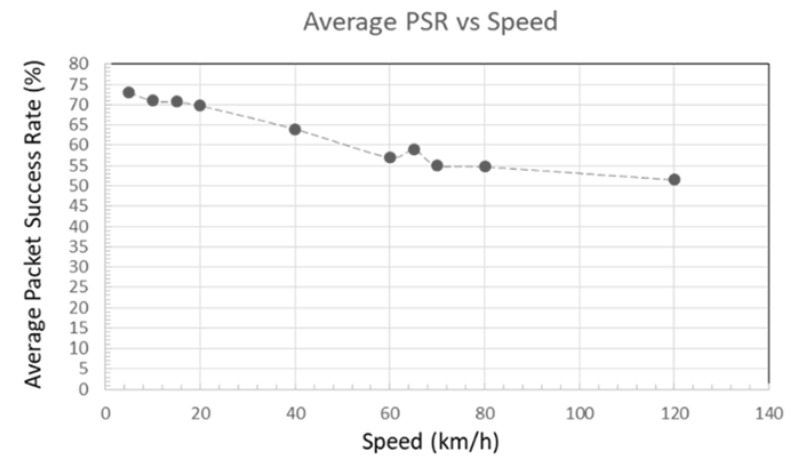
Average Packet Success Rate vs. Speed.

Figure 27 depicts, the influence of speed on the average packet success rate. It can be seen that the increased speed degrades the communication between the data mule and the data concentrator.

## 5. Discussion

**Figure 28:**
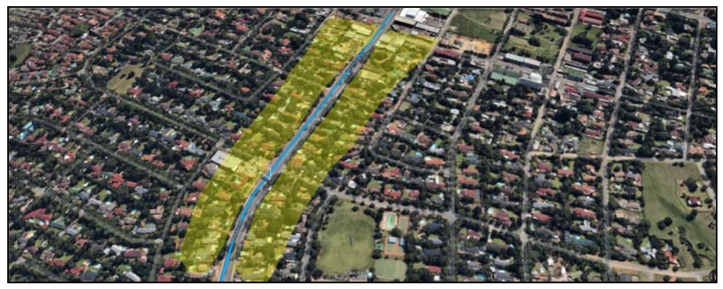
Coverage in Urban Area 60 km/h.

If we focus on packets with an 80% success rate, we can notice that at the lowest speed of 5 km/h, the data mule is able to collect data from a greater distance (172.56 m to be exact). At a speed of 60 km/h, which is the legal speed in residential areas, reliable data collection becomes possible when the taxi is 107.25 m away from the data collector. An illustration of the collection area at speed of 60 km/h is depicted in Figure 28.

At the fastest speed of 120 km/h (for highways), 80% success rate is possible 35.6 m after passing the data concentrator. At lower speed, the data mule has adequate time to packetize data and successfully transmit hence the data mule is able to sustain a high success rate for a longer distance. At higher speed, high packet success rate is only possible for a short duration (less than 17 s).

We also analyse the effect of different distance between the data concentrator and the road used by the data mule. On a section of the N1 highway that was surveyed, the distance between the centre of the road and the nearest habitation varied between 49 m and 59 m. In residential areas, the distance was more or less 18 m between the centre of the road and the boundary wall. Based on these various distances we mapped the relationship between the PSR and the distance between the data concentrator and the road. We noticed that the maximum distance to guarantee 80% PSR was 150 m. At that distance, 80% PSR was possible at speed lower than 60 km/h for a maximum 124 s.

When the mini-bus taxi is in an urban area, a path loss exponent of 4.0 was used. The average speed in this area was found to be between 11 km/h and 14 km/h during peak hours and 20 km/h without traffic. This average speed was obtained by only considering the time it took for a vehicle to travel between two points. Using the speed obtained, we assess the effectiveness of data collection of building of multiple height. Due to the increased interference in the environment, the 80% transmission window is 18 s for speeds less than 20 km/h and 9 s for greater speeds. The highest building able to transmit information to the data mule has been found to be 90 m tall. At that heights, for a speed less than 20 km/h, the transmission window will be less than 8 s.

Base on the result obtained, we can derive the following polynomial trend function to determine the coverage of a static data concentrator fitted with an XBee Pro in relation to the speed of data mule:

$$x=-0.0077v^{2}-0.793v+173.37,$$

with an error percentage of 1.8% under ideal circumstances.

Using the trend function above, we will be able to determine the minimum transmission radius of the data concentrator with respect to the speed limit of the nearby roads that the public transport will use. We will be able to optimally place the concentrator to maximize data collection.

For residential areas, based on the formula above, the concentrator should be placed at a distance less than 107 m from the road if an 18 dBm ZigBee transmitter is used. Furthermore, if we assume that the vehicle does not stop moving while in the communication range, the communication must take place within 17 s if the speed is less than 60 km/h in order to insure 80% reliability.

## 6. Conclusions

Our main objective in this research was to determine the parameters required to successfully implement a billing system for electricity that utilize data collected using a mini-bus taxi. From the multiple experiments conducted, key parameters and patterns describing the relationship between a data mule speed and the packet success rate have been discovered. Based on our work, a procedure to obtain the optimum position of a static data concentrator in relation to the speed limit of the road frequently used by the data mule was revealed. Our research also presented specific communication time constraint that applies to data mule under multiple environments. 

A detailed system design using new technology and readily available components was included in this work. One of the most popular internet of things device, the Raspberry Pi 3B was used in the design of our proposed billing and data collection solution.

By utilizing governmental Wi-Fi services or Wi-Fi connectivity at the taxi ranks, daily data upload to the main billing server could be possible. Under ideal circumstances and with a 67 bytes data packet per electricity meters, a mini-bus taxi data mule should be able to collect data from a maximum of 4000 electricity meters in less than 10 s if we assume minimal connection and packetization delay. This is due to the fact that the ZigBee data rate is 250 kilobits per second.

By using the Bluetooth capabilities of the data mule and a data collection mobile application, commuters or technicians on the bus will be able to upload metering data directly to the billing cloud server. We are hoping that the work conducted in our research will pave the way for a novel billing system that is both accurate and cost effective.

This work could form the backbone of a community data collection scheme that would enable anyone to collect smart meter data and submit it seamlessly to a cloud server while on their way to work. The person collecting smart meter data could be given a reward by government based on the amount of data collected.

In the future, we are hoping to extend our work to other form of transport such as trains. Future research could include the use of autonomous drones as data mules. Using multiple artificial intelligence techniques, a drone will be able to determine optimum collection path that will yield the highest data collection success rate. Multiple other low cost wireless communication protocols such as LoRa could also be explored in order to upload billing data. With the potential high amount of data to be collected by the billing system, data processing requiring a combination of real time database such as Firebase and big data processing techniques will need to be explored.
